# P-689. Racial and Ethnic Disparities in RSV Pneumonia: Propensity Score Matched Analysis from All of Us Cohort

**DOI:** 10.1093/ofid/ofaf695.902

**Published:** 2026-01-11

**Authors:** Sailesh Shrestha, Vel Sivapalan

**Affiliations:** NYC Health + Hospitals/Harlem, New York, NY; NYC Health + Hospitals/Harlem, New York, NY

## Abstract

**Background:**

Respiratory syncytial virus (RSV) is increasingly recognized as a significant cause of adult viral pneumonia, particularly among those with comorbid conditions. Understanding racial and ethnic disparities in RSV infection is crucial for targeted public health interventions, especially RSV vaccinations.

**Figure:**
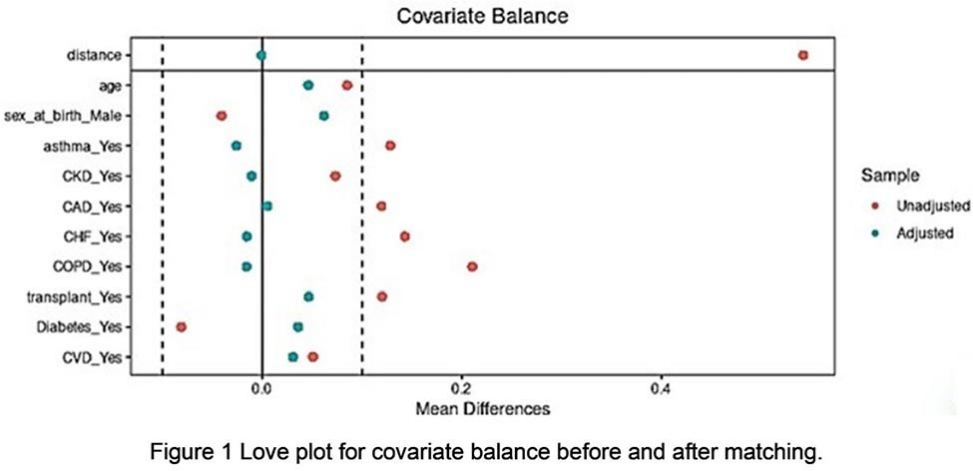

**Figure:**
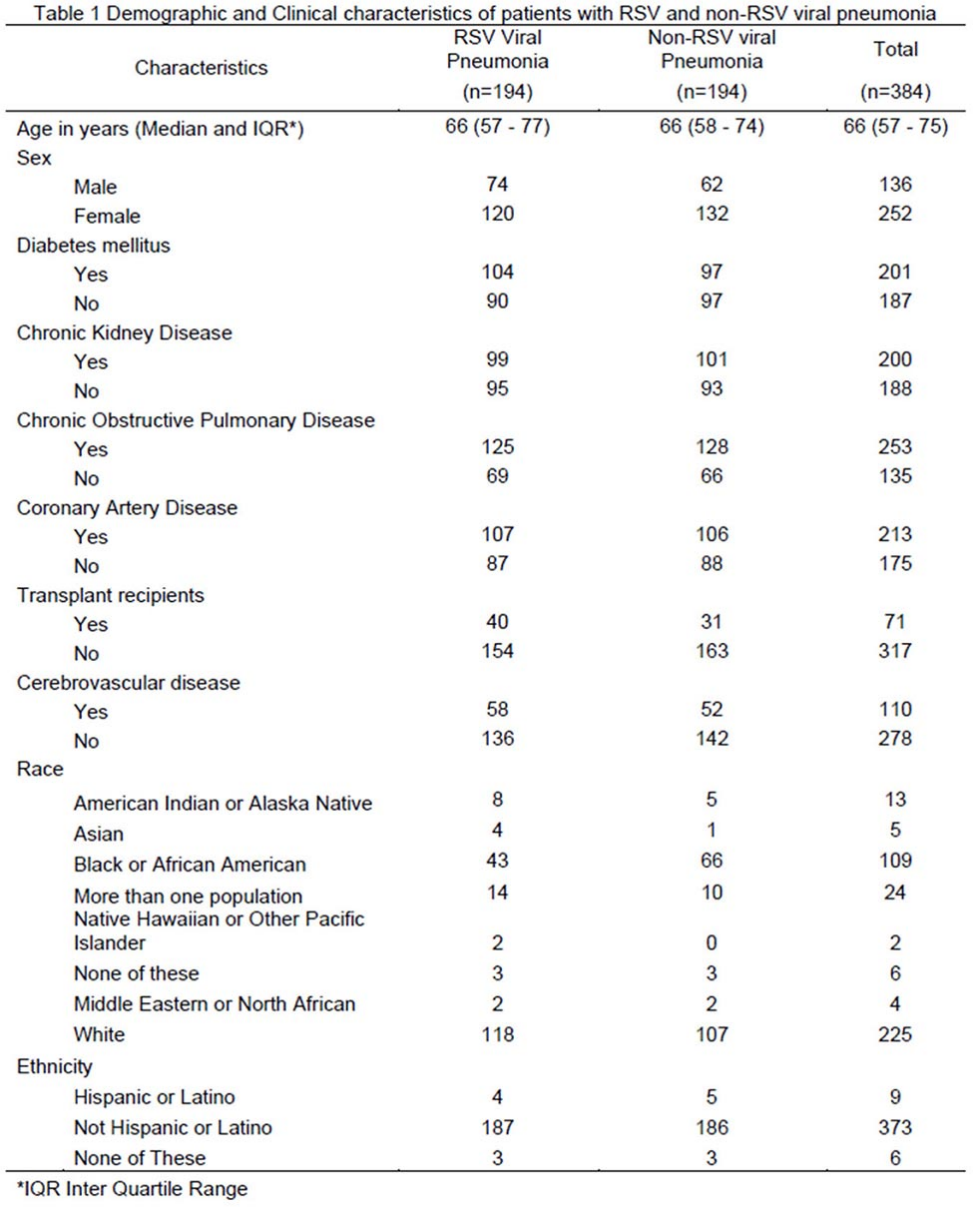

**Methods:**

We conducted a cross-sectional analysis of the All of Us Research controlled tier database version 8 within the researcher workspace. Adults aged ≥ 18 years with viral pneumonia (Concept ID 261326, SNOMED Code 755700040) were divided into RSV-positive cases (Concept ID 436145, SNOMED Code 195881003) and the rest as RSV-negative controls. We conducted a propensity score matching analysis between the cases and controls using logistic regression, adjusting for key covariates: age, sex at birth, asthma, chronic kidney disease, coronary artery disease, congestive heart failure, chronic obstructive pulmonary disease, organ transplant status, diabetes mellitus, and cerebrovascular disease. We assessed the covariate balance using standardized mean differences before and after matching with the Love plot. All subsequent analyses were performed using the matched sample. Ethnicity and race distributions between the cases and controls were then compared using chi-squared or Fisher’s Exact tests as appropriate.

**Figure:**
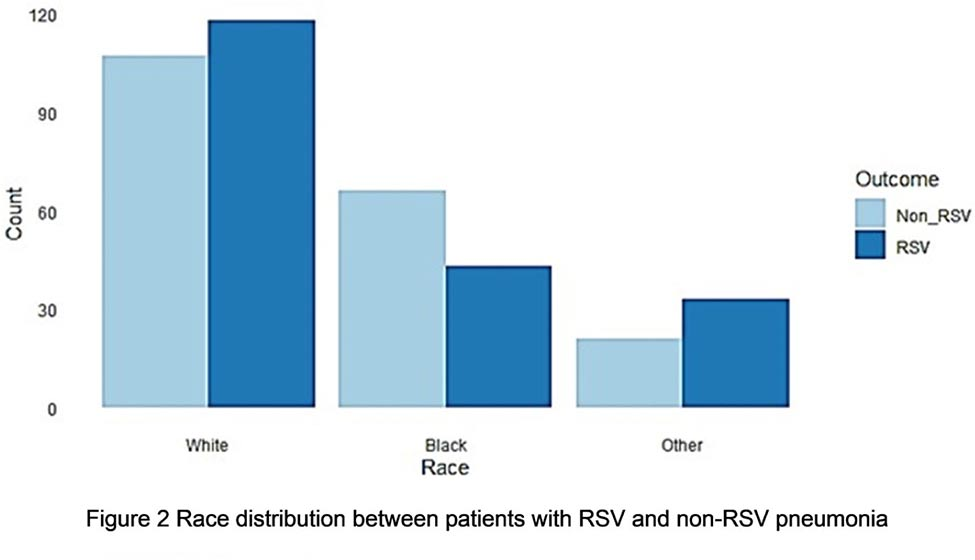

**Figure:**
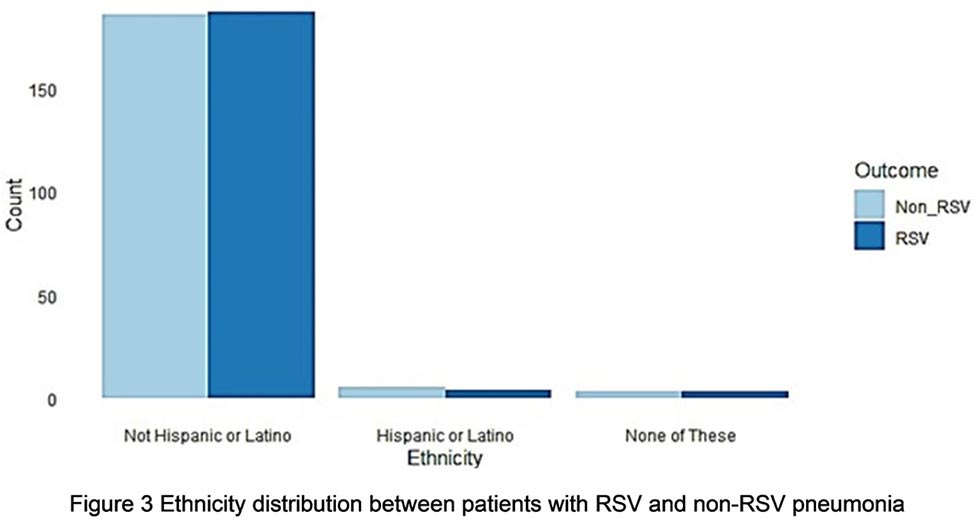

**Results:**

A total of 194 patients with RSV viral pneumonia were matched with an equal number of patients with non-RSV viral pneumonia, selected from 3589 controls, using nearest-neighbor matching without replacement (Table 1 and Figure 1). We regrouped the race into white, black, and other categories, and conducted a Pearson's Chi-squared test, which showed a statistically significant association between race and RSV pneumonia (p-value 0.0178, Figure 2). We conducted a Fisher's Exact Test, which indicated that there was no statistically significant relationship between ethnicity and RSV pneumonia (p-value 1, Figure 3).

**Conclusion:**

In this propensity score–matched analysis, adjusting for clinical risk factors, we found significant disparities on RSV pneumonia based on race but not ethnicity. Further research is needed to explore these disparities in the context of genetic, socioeconomic, or healthcare access factors.

**Disclosures:**

All Authors: No reported disclosures

